# Quantifying Gene Regulatory Relationships with Association Measures: A Comparative Study

**DOI:** 10.3389/fgene.2017.00096

**Published:** 2017-07-13

**Authors:** Zhi-Ping Liu

**Affiliations:** Department of Biomedical Engineering, School of Control Science and Engineering, Shandong University Jinan, China

**Keywords:** gene regulatory network, gene coexpression, association measure, high-throughput data, bioinformatics

## Abstract

In this work, we provide a comparative study of the main available association measures for characterizing gene regulatory strengths. Detecting the association between genes (as well as RNAs, proteins, and other molecules) is very important to decipher their functional relationship from genomic data in bioinformatics. With the availability of more and more high-throughput datasets, the quantification of meaningful relationships by employing association measures will make great sense of the data. There are various quantitative measures have been proposed for identifying molecular associations. They are depended on different statistical assumptions, for different intentions, as well as with different computational costs in calculating the associations in thousands of genes. Here, we comprehensively summarize these association measures employed and developed for describing gene regulatory relationships. We compare these measures in their consistency and specificity of detecting gene regulations from both simulation and real gene expression profiling data. Obviously, these measures used in genes can be easily extended in other biological molecules or across them.

## Introduction

The high-throughput technologies, such as microarray (Schena et al., 1995) and RNA-Seq (Wang et al., 2009) in transcriptomic level, generate bunch of data of describing various perspectives of cell state. These data provide unprecedented opportunity to quantify molecular expressions and their relationships. From a systematic perspective, the molecules in a cell orchestrate together to form various integrated and condense network systems of performing comprehensive functions (Liu, 2015). For instance, transcriptional interactions between transcription factor (TF) and target genes are often formulated into gene regulatory network of modeling biological processes (Liu et al., 2014, 2015). Deciphering gene relationships from high-throughput data are crucial to reversely engineer their inner interaction scenarios, as well as profoundly reveal the dysfunctions in certain disorders, such as complex diseases (Liu et al., 2012).

Quantifying the relationship between molecular components becomes fundamental in the new research paradigm from data to knowledge. The data analysis techniques of association support the kind of investigation. Traditionally, when we explore the relationship between two variables, Pearson's correlation coefficient (PCC) is employed to qualify their linear relationship (Zou et al., 2003). From entropy aspects, mutual information (MI) is often used for defining the non-linear relationship between gene variables (Butte and Kohane, 2000). Mathematically, the assumptions underlying these measures are considerable in real applications. Association measures have been developed to meet the requirements of appropriateness and precision in defining relationships from various perspectives.

Detecting gene associations is a fundamental method to reconstruct gene regulatory network from gene expression profiling data (Liu, 2015). Although more integrated methods such as ordinary differential equations are available to model the differential dynamics among genes, the association-based methods are direct, simple, and easy for interpretation as well. With introducing the independence, these measures have been extended to quantify the associations between many genes simultaneously (Stuart et al., 2003). In typical microarray experiments, the gene expression data can often be represented by matrix **G**,

$$\begin{array}{l} G=\left(\begin{array}{c} G_{1}\\ G_{2}\\ \vdots \\ G_{m} \end{array}\right)=\left(\begin{array}{ccccc} G_{11} & \ldots & G_{1j} & \ldots & G_{1n}\\ \vdots & \ddots & \vdots & \ddots & \vdots \\ G_{i1} & \ldots & G_{ij} & \ldots & G_{in}\\ \vdots & \ddots & \vdots & \ddots & \vdots \\ G_{m1} & \ldots & G_{mj} & \ldots & G_{mn} \end{array}\right). \end{array}$$

**Figure 1:**
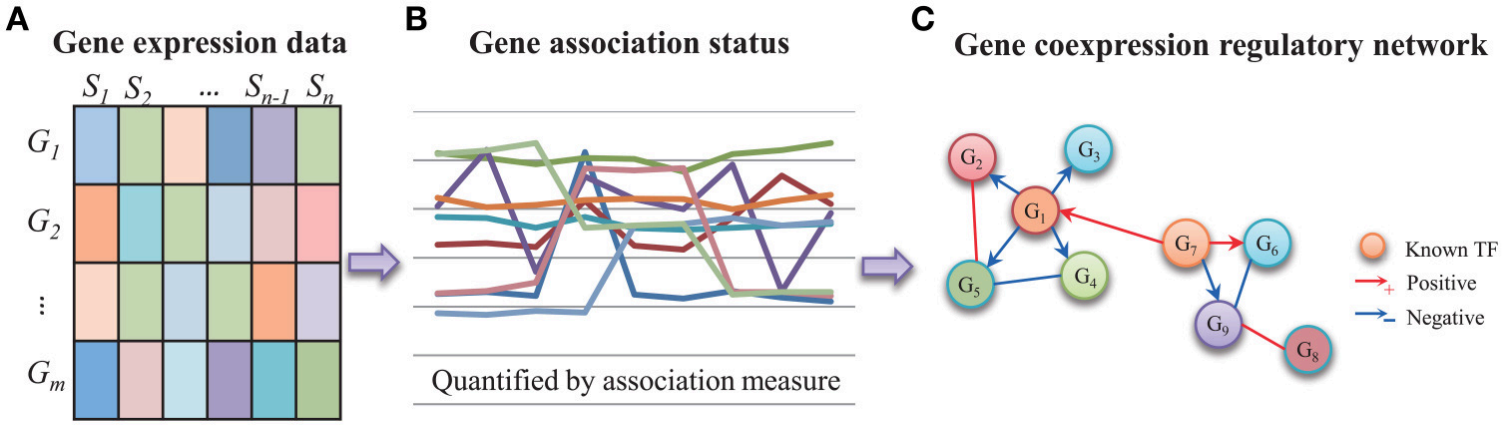
The strategy of building gene coexpression-based regulatory network from gene expression data. **(A)** The gene expression patterns of *m* genes in *n* samples. **(B)** The gene coexpression patterns quantified by association measure. **(C)** With some prior knowledge of TFs, the gene coexpression relationships can be improved to be a gene regulatory network.

Where *G*_*ij*_ represents the gene expression value of the *i*-th gene (1 ≤ *i* ≤ *m*) in the *j*-th experiment (1 ≤ *j* ≤ *n*). It is noted that *j* refers to a sample or a time point with specific phenotype meaning. The association between gene *X* and gene *Y* (*X, Y* ∈ {*G*_1_, *G*_2_, ⋯ , *G*_*m*_}) is often to indicate their regulatory relationship (Zhang and Horvath, 2005). Let gene expressions be *X* = (*X*_1_, *X*_2_, …, *X*_*n*_) and *Y* = (*Y*_1_, *Y*_2_, …, *Y*_*n*_). Based on the two vectors, we employ or define an association measure to assess their regulatory strength. Recently, some novel measures besides PCC and MI have been proposed to define the association between two variables (Reshef et al., 2011). It is of great interest to investigate their performances in the reconstruction of gene regulatory network from gene expression data. Figure 1 demonstrates the strategy of inferring gene regulatory network by gene coexpression analysis. Gene regulation, in a particular form of transcriptional regulation, often specifies the regulation from TF to target gene. The quantified gene coexpression evaluates the simultaneous patterns of two gene's redundancy across samples. The expression level of upstream TF's gene is often to approximate its downstream protein product. As shown in Figure 1C, if we set up which ones are TFs by prior knowledge in the gene association network, we can infer a directed gene regulatory network via an undirected association measure.

The coexpression pattern between two genes implies their regulatory aspects. As shown in Figure 1C, it firstly indicates a direct regulatory interaction. In some biological state, gene coexpression exactly responds to the activation or inhibition regulation from a TF to its target gene. The regulation between them is reflected by their highly-related gene expression redundancy. Secondly, gene coexpression is about gene co-regulation. That is to imply the two genes are regulated by the same TF(s) and then they contain highly-related gene expression patterns. Third means that the two genes are functionally-related by participating in the same regulatory circuit or particular signaling pathway. Generally, the dynamic regulations in a cell are inherently embedded with temporal features. Gene regulation is often reflected by time-delayed gene expression patterns from the activation of TF's gene to the downstream target responds (Bar-Joseph et al., 2012). For the simplicity of association measure, the coexpression-based methods are popular in inferring gene regulatory network from gene expression data (Zhang and Horvath, 2005).

In this paper, we provide a comparative study on these available association measures of quantifying gene relationships in regulatory network. Fourteen most-popular association measures or indices will be summarized and compared. Based on some benchmark datasets of gene regulatory network inference challenges, we evaluate their individual performances in the reconstruction of gene regulatory networks. This provides a concise comparison of accuracy and quality in network inference by the association measures. In a case study, we compare the differences of these inferred regulations during the infection of hepatitis C virus on host cells. In data-driven network inference, the characteristics of the association measures in statistics and computations are also analyzed and discussed.

## Association measures

Numerous association measures have been proposed to define the relationship between two random variables. For gene regulations, we collect 14 of them for our assessments of network inference power from data. Table 1 lists the 14 association measures with brief introduction of their statistical assumptions and fundamental properties individually. Some measures are well-known such as PCC, while some become available recently such as maximal information correlation (MIC). For the completeness of introduction and reference, we describe them in details respectively in this section.

**Table 1:** Summary of some association measures used to quantify gene regulations.

**Abbre**.	**Method**	**Symbol**	**Description**	**References**
Pearson	Pearson's	*r*	Linear, widely-used, no parameter, coeff. ∈ [−1, 1]	Pearson, 1895
Spearman	Spearman's	ρ	Monotonic, rank-based, no parameter, coeff. ∈ [−1, 1]	Spearman, 1904
Kendall	Kendall's	τ	Monotonic, rank-based, no parameter, coeff. ∈ [−1, 1]	Kendall, 1938
Hoeffding	Hoeffding's	*D*	Non-linear, rank-based, no parameter, coeff. ∈ [0, 1]	Hoeffding, 1948
Blomqvist	Blomqvist's	β	Monotonic, rank-based, no parameter, coeff. ∈ [−1, 1]	Blomqvist, 1950
Goodman	Goodman and Kruskal's	γ	Monotonic, cross classifications, rank-based, no parameter, coeff. ∈ [−1, 1]	Goodman and Kruskal, 1954
WWH	Wang, Waterman, Huang's	*wwh*	Monotonic, rank-based, no parameter, coeff. ∈ [0, +∞]	Wang et al., 2014
MI	Mutual information	*I*	Non-linear, entropy-based, no parameter, coeff. ∈ [0, +∞]	Shannon, 1948
MIC	Maximum information correlation	*mic*	Non-linear, entropy-based, 1 parameter, coeff. ∈ [0, 1]	Reshef et al., 2011
Wilks	Wilks'	*W*	Linear, covariance-based, no parameter, coeff. ∈ [0, 1]	Wilks, 1935
KCCA	Kernel canonical correlation analysis	*kcca*	Non-linear, covariance-based, 1 parameter, coeff. ∈ [0, 1]	Bach and Jordan, 2002
dCor	Distance correlation	*dCor*	Non-linear, covariance-based, 1 parameter, coeff. ∈ [0, 1]	Szekely and Rizzo, 2009
CMMD	copula-based maximum mean discrepancy	*cmmd*	Non-linear, copulas-based, 1 parameter, coeff. ∈ [0, 1]	Poczos et al., 2012
RDC	Randomized dependence coefficient	*rdc*	Non-linear, copulas-based, 2 parameters, coeff. ∈ [0, 1]	Lopez-Paz et al., 2013

### Pearson's correlation coefficient

PCC describes the linear relationship between two variables *X* and *Y* (Pearson, 1895). In the microarray data of gene expression, it defines the correlation coefficient between gene *X* and *Y* as

$$\begin{array}{l} r\left(X,Y\right)=\frac{\overset{n}{\underset{i=1}{\sum }}\left(X_{i}-\overset{-}{X}\right)\left(Y_{i}-Ȳ\right)}{\left(n-1\right)S_{X}S_{Y}}, \end{array}$$

where $\overset{-}{X}=\overset{n}{\underset{i=1}{\sum }}X_{i}$, $Ȳ=\overset{n}{\underset{j=1}{\sum }}Y_{j}$ refer to the mean of two variables of gene expression in samples, and $S_{X}=\sqrt{\frac{\overset{n}{\underset{i=1}{\sum }}{\left(X_{i}-\overset{-}{X}\right)}^{2}}{n-1}}$, $S_{Y}=\sqrt{\frac{\overset{n}{\underset{j=1}{\sum }}{\left(Y_{j}-Ȳ\right)}^{2}}{n-1}}$ are their standard deviations. Generally, it assesses their linear relationship into a value between −1 and 1, where 1 refers to total positive correlation and −1 refers to total negative correlation, and 0 refers to no correlation.

When we implement the statistical test of its significance, PCC assumes the two variables are from two normal distributions and the two vectors are the corresponding pairs with independence in the observations (Zou et al., 2003). It has been widely used to quantify the gene coexpression relationships in many studies, such as WGCNA (Zhang and Horvath, 2005; Langfelder and Horvath, 2008).

### Spearman's rank correlation

Spearman's rank correlation ρ is a non-parametric measure of the relationship between two variables (Spearman, 1904). The association between two variables *X* and *Y* is formulated as a monotonic function

$$\begin{array}{l} \rho =1-\frac{6\overset{n}{\underset{i=1}{\sum }}d_{i}^{2}}{n\left(n^{2}-1\right)}. \end{array}$$

Where *d*_*i*_ = *X*_*i*_ − *Y*_*i*_, 1 ≤ *i* ≤ *n*. Instead of using the element values directly, it transforms the two vectors to the two rank vectors of these elements respectively. The differential rank vector is generated by the difference between two rank vectors.

When there are no repeated values in *X* and *Y* (no duplicated ranks), ρ reaches 1 and −1 when a variable is a perfect monotone function of the other variable. The statistical independence between them refers to ρ = 0. In the statistical test, it still requires the dependence between the two ranking of two variables (Zar, 1972). Compared to PCC, it contains a larger application scope because it does not require the normal distribution assumptions. It is equivalent to PCC between two ranked variables (Conover and Iman, 1981). The following non-linear rank-based correlations contain the similar properties.

### Kendall's tau coefficient

Similar to the former coefficients, Kendall's tau coefficient (Kendall, 1938) is another measure of rank correlation between *X* and *Y*. It is defined as

$$\begin{array}{l} \tau =\frac{n_{c}-n_{d}}{n\left(n-1\right)/2}, \end{array}$$

where *n*_*c*_ = #(*concordantpairs*) and *n*_*d*_ = #(*discordantpairs*). Any pair of observations (*X*_*i*_, *Y*_*i*_) and (*X*_*j*_, *Y*_*j*_) in *X* and *Y*, where *i* ≠ *j*, are defined as concordant if the ranks for both elements agree, i.e., if both *X*_*i*_ > *X*_*j*_ and *Y*_*i*_ > *Y*_*j*_ or if both *X*_*i*_ < *X*_*j*_ and *Y*_*i*_ < *Y*_*j*_. They are classified to be discordant if *X*_*i*_ > *X*_*j*_ and *Y*_*i*_ < *Y*_*j*_ or if *X*_*i*_ < *X*_*j*_ and *Y*_*i*_ > *Y*_*j*_. If *X*_*i*_ = *X*_*j*_ or *Y*_*i*_ = *Y*_*j*_, the pair is neither concordant nor discordant. Based on τ, Somers' *D* of *Y* with respect to *X* is defined as *D*_*YX*_ = τ(*X, Y*)/τ(*X, X*), where τ(*X, X*) is the number of pairs with unequal values (Somers, 1962). It is easy to find that the order of ranks in the two variables plays critical roles in the calculation of these non-parametric estimators.

### Hoeffding's dependence coefficient

The original idea of Hoeffding's dependence measure *D* is to assess the independence of two datasets by their distance between distributions for continuous variables (Hoeffding, 1948). It has been extended for the samples of *X* and *Y* as

$$\begin{array}{l} D=\frac{\left(n-2\right)\left(n-3\right)D_{1}+D_{2}-2\left(n-2\right)D_{3}}{n\left(n-1\right)\left(n-2\right)\left(n-3\right)\left(n-4\right)}, \end{array}$$

where $D_{1}=\underset{i}{\sum }\left(Q_{i}-1\right)\left(Q_{i}-2\right)$, $D_{2}=\underset{i}{\sum }\left(R_{i}-1\right)\left(R_{i}-2\right)\left(S_{i}-1\right)\left(S_{i}-2\right)$ and $D_{3}=\underset{i}{\sum }\left(R_{i}-2\right)\left(S_{i}-2\right)\left(Q_{i}-1\right)$, *R*_*i*_ is the rank of *X*_*i*_, *S*_*i*_ is the rank of *Y*_*i*_, and *Q*_*i*_ is the bivariate rank, which refers to the number of points with both *X* and *Y* values less than the *i*th point, i.e., *Q*_*i*_ = #(*X*_*j*_, *Y*_*j*_) *s.t*. *X*_*j*_ < *X*_*i*_ *and Y*_*j*_ < *Y*_*i*_.

### Blomqvist's β

A measure referred as Blomqvist's β has been developed for the medial correlation coefficient (Blomqvist, 1950). For two random variables *X* and *Y*, let “*x* − *y*”-plane be divided into four regions by the median lines of $\tilde{x}$ and ỹ. The relationship of *X* and *Y* can be obtained from the number of sample points in the four quadrants. In gene regulations, suppose the sample size takes even number (with minor modifications in odd number), it is defined as

$$\begin{array}{l} \beta =\frac{n_{1}-n_{2}}{n_{1}+n_{2}}=\frac{2n_{1}}{n_{1}+n_{2}}-1, \end{array}$$

where *n*_1_ refers to the number of data in the first or third quadrant, and *n*_2_ refers to that in the second or fourth quadrant. It has some advantages such as its explicit form and low computational complexity in estimation (Blomqvist, 1950).

### Goodman and Kruskal's gamma coefficient

The Goodman and Kruskal's γ coefficient (Goodman and Kruskal, 1954) is another widely-used rand-based coefficient to measure the dependence between variables. It is defined as

$$\begin{array}{l} \gamma =\frac{P_{s}-P_{d}}{P_{s}+P_{d}}, \end{array}$$

where *P*_*s*_, *P*_*d*_ are the probabilities that a randomly selected pair of observations will relocate in the same or opposite order respectively, when ranked by both variables. It represents the symmetric distances between the two paired sets representing the binary relation of ranks. It is very close to Kendall's tau. In gene samples, its maximum likelihood estimation can be regarded as

$$\begin{array}{l} G=\frac{n_{s}-n_{d}}{n_{s}+n_{d}}, \end{array}$$

where *n*_*s*_ is the number of concordant pairs, which refer to those pairs ranked in the same order one both variables. *n*_*d*_ is the number of discordant pairs, which are the number of pairs of cases ranked in reversed order. It computes the normalized difference between the numbers of concordant and discordant pairs such that it will take values between −1 and +1. When it is specified into 2 × 2 matrices, it is exactly Yule's *Q* coefficient (Yule, 1900).

### WWH order correlation

The order statistics seems to provide a robust gene coexpression measure by taking local patterns in gene expression profiles into account. Wang, Huang, and Waterman (WWH; Wang et al., 2014) proposed a count statistics method to define a new gene coexpression regulatory measure, i.e.,

$$\begin{array}{l} wwh=\underset{1\leq i_{1}<\cdots <i_{k}\leq n}{\sum }F\left(X_{i_{1}},\ldots ,X_{i_{k}};Y_{i_{1}},\ldots ,Y_{i_{k}}\right). \end{array}$$

Where *X* = (*X*_1_, …, *X*_*n*_) and *Y* = (*Y*_1_, …, *Y*_*n*_) are genes *X* and *Y* with expression levels from *n* samples. The function *F* is an indicator function comparing the rank patterns of the two subsequences with a length parameter *k*. This method aims to identify the consistency of rank orders of the two variables and expect to highlight the local corresponding features in expression profiles. The authors considered a special case in the time-series samples by constraining the consecutive subsequences and another general cases of samples (Wang et al., 2014).

### Mutual information

Mutual information is based on information theory (Shannon, 1948). Suppose *P*(*X, Y*) is the joint probability distribution function of gene variables of *X* and *Y*, and *P*(*X*) and *P*(*Y*) are their marginal probability distribution functions respectively. The mutual information between *X* and *Y* is defined as

$$\begin{array}{l} I=-\underset{X_{i}\in X,Y_{j}\in Y}{\sum }P\left(X_{i},Y_{j}\right)\log\frac{P\left(X_{i},Y_{j}\right)}{P\left(X_{i}\right)P\left(Y_{j}\right)}. \end{array}$$

The mutual information can also be represented as a Kullback–Leibler divergence (Kullback and Leibler, 1951), which is to measure of the difference between two probability distributions.

### Maximal information correlation

Based on mutual information, MIC is defined to evaluate the margin probability by calculating the data point frequencies (Reshef et al., 2011), i.e.,

$$\begin{array}{l} MIC=\underset{|X_{i}||Y_{j}|<\;B}{\max}\frac{I\left(X,Y\right)}{\log_{2}\left(\min\left(\left|X_{i}|,|Y_{j}\right|\right)\right)}, \end{array}$$

where (*X*_*i*_) and (*Y*_*j*_) are the two gene expressions across the samples individually. *I* refers to their mutual information. The *B* is a heuristically setting parameter such as *B* = *N*^0.6^, and *N* is the cells of a grid *G* induced by *X* and *Y*.

### Wilks' *W*

Wilks' *W* statistic is the covariance-based measure of two vectors (Wilks, 1935). It is defined as

$$\begin{array}{l} W=1-\frac{\det\left(\sum \right)}{\det\left(\sum_{11}\right)\det\left(\sum_{22}\right)}, \end{array}$$

where $\Sigma =\left(\begin{array}{cc} \Sigma_{11} & \Sigma_{12}\\ \Sigma_{21} & \Sigma_{22} \end{array}\right)$, and Σ_*ij*_ = cov(*X*_*i*_, *Y*_*j*_). It has close relationship with likelihood-ratio and multivariate analysis of variance (MANOVA) by integrating the covariances of two individual variables and their combinations. Similarly, Pillai's trace criterion performs similar ideas while with low popularity (Pillai, 1955). Here, it is a special case only for two gene expression vectors.

### Kernel canonical correlation analysis

Instead of directly calculating the relationship between *X* and *Y*, the canonical correlation analysis (CCA) is a statistical technique of maximizing the correlation between sets of projections of the two original vectors.

Let *U* = *a*^*T*^*X*, *V* = *b*^*T*^*Y*, $Var\left(U\right)=a^{T}\sum_{11}a$, $Var\left(V\right)=b^{T}\sum_{22}b$, $Cov\left(U,V\right)=a^{T}\sum_{12}b$,

where $\Sigma \;=Var(X,Y)=\left(\begin{array}{cc} \Sigma_{11} & \Sigma_{12}\\ \Sigma_{21} & \Sigma_{22} \end{array}\right)$, Σ_11_ = *Var*(*X*), Σ_22_ = *Var*(*Y*), Σ_12_ = *Var*(*X, Y*), Σ_21_ = *Var*(*Y, X*).

So

$$\begin{array}{l} Cor\left(U,V\right)=\frac{a^{T}\sum_{12}b}{\sqrt{a^{T}\sum_{11}a}\sqrt{b^{T}\sum_{22}b}}. \end{array}$$

We define the largest canonical correlation as $\rho_{1}=\underset{a,b}{\sup}\;Cor\left(U,V\right)$, where we set the second floor as a fix number. When we maximize the first floor by solving an optimization problem is to achieve the largest canonical correlation coefficient between the original *X* and *Y*.

In CCA, the vector of *U* and *V* are linear combinations of *X* and *Y*. When

$$\begin{array}{l} K_{X}=\sum_{i}\Phi {\left(X_{i}\right)}^{T}\Phi \left(X_{i}\right),\\ K_{Y}=\sum_{i}\Phi {\left(Y_{i}\right)}^{T}\Phi \left(Y_{i}\right), \end{array}$$

where Φ : ℝ^*n*^ → ℝ^*N*^(*n* ≤ *N*) is the kernel function of *X* and *Y* (can be different for them).

$$\begin{array}{l} Cor\left(U,V\right)=\frac{\alpha^{T}K_{X}K_{Y}\beta }{\sqrt{\alpha^{T}K_{X}K_{Y}\alpha }\sqrt{\alpha^{T}K_{X}K_{Y}\beta }}, \end{array}$$

and the kernel CCA is defined as $kcca\left(X,Y\right)=\underset{\alpha ,\beta }{\sup}\;Cor\left(U,V\right)$.

### Distance correlation

Let (*X*_*i*_, *Y*_*i*_), 1 ≤ *i* ≤ *n* be statistical samples for two random variables (*X, Y*). The pairwise distances are

$$\begin{array}{l} \begin{array}{c} a_{j,k}=||X_{j}-X_{k}||,j,k=1,2,\ldots ,n,\\ b_{j,k}=||Y_{j}-Y_{k}||,j,k=1,2,\ldots ,n, \end{array} \end{array}$$

where ||▪|| denotes Euclidean norm, Then, two *n* × *n* distance matrices (*a*_*j,k*_) and (*b*_*j,k*_) are generated. For each element (*j, k*), two transformed values are defined as

$$\begin{array}{l} A_{j,k}=a_{j,k}-{ā}_{j,▪}-{ā}_{▪,k}+{ā}_{▪,▪},\\ B_{j,k}=b_{j,k}-{\overset{-}{b}}_{j,▪}-{\overset{-}{b}}_{▪,k}+{\overset{-}{b}}_{▪,▪}, \end{array}$$

where ā_*j*,▪_ is the *j*-th row mean, ā_▪,*k*_ is the *k*-th column mean, and ā_▪,▪_ is the grand mean of the distance matrix of the *X* samples. The notations for *b* values have the similar meanings. The distance covariance is defined as the square root of

$$\begin{array}{l} V_{XY}^{2}=\frac{1}{n^{2}}\overset{n}{\underset{i,j=1}{\sum }}A_{i,j}B_{i,j}. \end{array}$$

Then, distance correlation (dCor; Szekely and Rizzo, 2009) between *X* and *Y* is defined as the square root of

$$\begin{array}{l} dCor=R^{2}=\frac{V_{XY}^{2}}{V_{X}V_{Y}}. \end{array}$$

dCor satisfies 0 ≤ *R* ≤ 1, and *R* = 0 when *X* and *Y* are independent.

### Copula-based maximum mean discrepancy

A copula is a multivariate probability distribution function defined on the unit hypercube with known uniform marginals (Nelsen, 2006). It is popular in high-dimensional statistics for describing the relationships between variables. Specifically, the copula of two random gene variables *X* and *Y* is defined as a function

$$\begin{array}{l} C\left(U,V\right)=C\left(F_{X}\left(x\right),F_{Y}\left(y\right)\right)=F_{XY}\left(x,y\right), \end{array}$$

where *F*_*X*_(*x*) = *P*(*X* ≤ *x*), *F*_*Y*_(*x*) = *P*(*Y* ≤ *y*), and *F*_*XY*_(*x, y*) = *P*(*X* ≤ *x, Y* ≤ *y*) are the two marginal distributions and the joint distributions (Sklar, 1959).

cMMD is a copula-based kernel association measure between random variables (Poczos et al., 2012). It extends the maximum mean discrepancy (MMD) method (Borgwardt et al., 2006) of measuring dependence to the copula of the joint distribution. Suppose two copulas transformations have been implemented on the original variables, i.e., *U* = *F*_1_(*X*) and *V* = *F*_2_(*Y*), *F*_1_ and *F*_2_ are the empirical cumulative distribution functions for *X* and *Y* respectively (Lopez-Paz et al., 2013). cMMD defines the relationship between *X* and *Y* as

$$\begin{array}{l} cmmd\;\left(X,Y\right)=mmd\left[F_{1}\left(X\right),F_{2}\left(Y\right)\right]=\frac{1}{n\left(n-1\right)}\overset{n}{\underset{i\neq j}{\sum }}K\left(U_{i},V_{j}\right), \end{array}$$

where *K*(*U*_*i*_, *V*_*j*_) = Φ(*U*_*i*_, *U*_*j*_) + Φ(*V*_*i*_, *V*_*j*_) − Φ(*U*_*i*_, *V*_*j*_) − Φ(*U*_*j*_, *V*_*i*_), and Φ is a specified kernel function, e.g., Gaussian kernel.

### Randomized dependence coefficient

Based on the former kernel CCA and copulas, the randomized dependence coefficient (RDC) provides a computationally efficient association measures between multivariate random variables. In details, it is defined as

$$\begin{array}{l} rdc\left(X,Y;k,s\right)=\underset{\alpha ,\beta }{\sup}Cor\left\{\alpha^{T}\Phi \left[F_{1}\left(X\right);k,s\right],\;\beta^{T}\Phi \left[F_{2}\left(Y\right);k,s\right]\right\}, \end{array}$$

where the functions are the same as the former ones, *k* ∈ ℕ^+^ and *s* ∈ ℝ^+^ are the parameters which are often set as 20 and 0.6 respectively. RDC is proved to be capable of discovering a wide range of functional association patterns in multiple datasets.

## Results of comparison study

**Figure 2:**
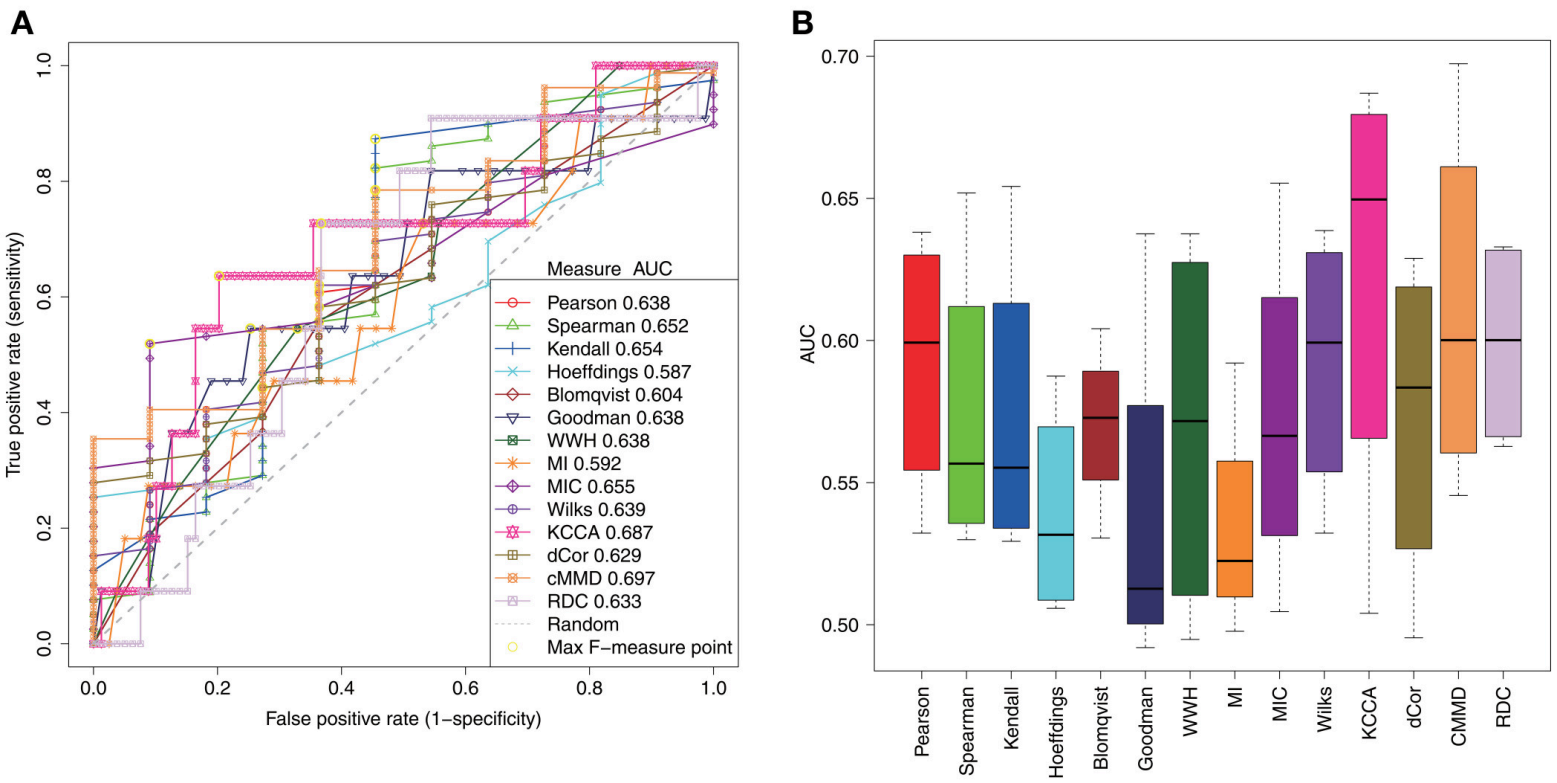
The performances of different association measures in the inference of the 10-node regulatory network of DREAM challenges. **(A)** ROC curve of 14 association measures with maximum AUC in the four datasets. **(B)** Blox plots of AUC of 14 association measures.

For a comparative study of these association measures in inferring gene regulatory relationships, we test these association measures in DREAM3 *in silico* network challenge datasets (Marbach et al., 2010). In the challenges, gene expression datasets have been generated by some specified network structures. Then, the datasets are open without any information about the network structures. The task is to reconstruct the network structures from the open datasets by developing new inference methods. There are three sizes of networks with 10, 50, and 100 nodes respectively, and multiple datasets for each size (4 for 10-node network, 23 for 50-node network, and 46 for 100-node network). The assessment is to evaluate the consistency between the inferred network and the true network structure (gold standards). Figure 2 illustrate the receiver operating characteristic (ROC) curves of inference performance by these association measures in the 10-node benchmark network. Due to the undirected regulations identified by all these association measures, we omit the regulatory directions when calculating the evaluation metrics of sensitivity (SN), specificity (SP), accuracy (ACC), Matthews correlation coefficient (MCC), F-measure, and area under ROC curve (AUC). Table 2 demonstrates these detailed values of evaluation metrics of these association measures. We find KCCA performs the best in the 14 association measures for inferring 10-node networks and it reaches the AUC of 0.623 ± 0.083 (mean ± standard deviation). Overall, the performances of these methods are comparable with each other in the 10-node network.

**Table 2:** The performance details of inferring benchmark gene regulatory networks by 14 association measures.

**Methods**	**Node size**	**SN**	**SP**	**ACC**	**F-measure**	**MCC**	**AUC**
Pearson	10	0.500 ± 0.093	0.545 ± 0.166	0.506 ± 0.098	0.518 ± 0.121	0.030 ± 0.162	0.592 ± 0.048
	50	0.536 ± 0.102	0.510 ± 0.121	0.535 ± 0.099	0.507 ± 0.074	0.014 ± 0.044	0.554 ± 0.027
	100	0.531 ± 0.047	0.487 ± 0.078	0.530 ± 0.046	0.504 ± 0.048	0.004 ± 0.021	0.536 ± 0.021
Spearman	10	0.617 ± 0.162	0.477 ± 0.155	0.600 ± 0.150	0.526 ± 0.141	0.074 ± 0.191	0.574 ± 0.055
	50	0.511 ± 0.083	0.504 ± 0.071	0.510 ± 0.081	0.502 ± 0.059	0.005 ± 0.036	0.538 ± 0.031
	100	0.501 ± 0.055	0.506 ± 0.086	0.501 ± 0.053	0.497 ± 0.043	0.002 ± 0.019	0.533 ± 0.025
Kendall	10	0.601 ± 0.192	0.500 ± 0.117	0.589 ± 0.175	0.536 ± 0.125	0.082 ± 0.198	0.574 ± 0.057
	50	0.499 ± 0.098	0.518 ± 0.083	0.500 ± 0.095	0.498 ± 0.053	0.005 ± 0.034	0.536 ± 0.031
	100	0.509 ± 0.054	0.503 ± 0.085	0.509 ± 0.053	0.499 ± 0.040	0.003 ± 0.017	0.532 ± 0.025
Hoeffdings	10	0.519 ± 0.591	0.591 ± 0.091	0.528 ± 0.080	0.544 ± 0.042	0.073 ± 0.062	0.539 ± 0.039
	50	0.507 ± 0.072	0.494 ± 0.102	0.507 ± 0.070	0.492 ± 0.064	0.00006 ± 0.038	0.544 ± 0.032
	100	0.504 ± 0.071	0.523 ± 0.061	0.504 ± 0.069	0.508 ± 0.042	0.006 ± 0.018	0.535 ± 0.025
Blomqvist	10	0.563 ± 0.069	0.409 ± 0.189	0.544 ± 0.060	0.451 ± 0.136	−0.019 ± 0.125	0.570 ± 0.030
	50	0.457 ± 0.126	0.496 ± 0.134	0.458 ± 0.120	0.444 ± 0.069	−0.016 ± 0.028	0.535 ± 0.030
	100	0.550 ± 0.066	0.583 ± 0.056	0.551 ± 0.065	0.560 ± 0.020	0.030 ± 0.008	0.574 ± 0.022
Goodman	10	0.411 ± 0.130	0.500 ± 0.053	0.422 ± 0.073	0.437 ± 0.073	−0.063 ± 0.073	0.539 ± 0.067
	50	0.470 ± 0.086	0.454 ± 0.083	0.469 ± 0.082	0.448 ± 0.037	−0.0246 ± 0.0194	0.531 ± 0.026
	100	0.531 ± 0.068	0.529 ± 0.059	0.531 ± 0.067	0.524 ± 0.027	0.014 ± 0.011	0.527 ± 0.018
WWH	10	0.411 ± 0.248	0.591 ± 0.174	0.433 ± 0.200	0.416 ± 0.148	−0.006 ± 0.103	0.569 ± 0.069
	50	0.352 ± 0.116	0.660 ± 0.099	0.360 ± 0.111	0.437 ± 0.083	0.003 ± 0.019	0.532 ± 0.016
	100	0.392 ± 0.137	0.619 ± 0.145	0.395 ± 0.134	0.442 ± 0.070	0.003 ± 0.009	0.522 ± 0.018
MI	10	0.557 ± 0.149	0.409 ± 0.241	0.539 ± 0.111	0.416 ± 0.115	−0.022 ± 0.111	0.534 ± 0.041
	50	0.470 ± 0.100	0.443 ± 0.081	0.470 ± 0.098	0.448 ± 0.069	−0.028 ± 0.043	0.569 ± 0.046
	100	0.468 ± 0.081	0.471 ± 0.069	0.468 ± 0.079	0.462 ± 0.046	−0.014 ± 0.020	0.544 ± 0.034
MIC	10	0.500 ± 0.051	0.636 ± 0.196	0.517 ± 0.042	0.547 ± 0.066	0.090 ± 0.121	0.573 ± 0.062
	50	0.515 ± 0.120	0.494 ± 0.084	0.515 ± 0.116	0.492 ± 0.070	0.003 ± 0.044	0.551 ± 0.031
	100	0.510 ± 0.058	0.502 ± 0.071	0.510 ± 0.057	0.501 ± 0.038	0.003 ± 0.017	0.531 ± 0.024
Wilks	10	0.522 ± 0.113	0.477 ± 0.087	0.517 ± 0.109	0.498 ± 0.098	0.0004 ± 0.13	0.592 ± 0.048
	50	0.536 ± 0.102	0.509 ± 0.120	0.536 ± 0.099	0.507 ± 0.073	0.014 ± 0.044	0.554 ± 0.027
	100	0.523 ± 0.050	0.502 ± 0.080	0.523 ± 0.049	0.508 ± 0.048	0.006 ± 0.021	0.538 ± 0.025
KCCA	10	0.472 ± 0.267	0.432 ± 0.202	0.467 ± 0.231	0.393 ± 0.168	−0.067 ± 0.219	0.623 ± 0.083
	50	0.442 ± 0.121	0.464 ± 0.119	0.442 ± 0.117	0.428 ± 0.070	−0.031 ± 0.037	0.541 ± 0.058
	100	0.453 ± 0.100	0.502 ± 0.090	0.454 ± 0.098	0.462 ± 0.058	−0.011 ± 0.024	0.541 ± 0.036
dCor	10	0.506 ± 0.061	0.545 ± 0.166	0.511 ± 0.069	0.520 ± 0.102	0.034 ± 0.140	0.573 ± 0.060
	50	0.529 ± 0.084	0.513 ± 0.103	0.529 ± 0.082	0.512 ± 0.067	0.014 ± 0.042	0.556 ± 0.031
	100	0.514 ± 0.061	0.510 ± 0.091	0.514 ± 0.060	0.505 ± 0.049	0.006 ± 0.021	0.538 ± 0.025
CMMD	10	0.573 ± 0.201	0.545 ± 0.129	0.569 ± 0.176	0.540 ± 0.112	0.085 ± 0.164	0.611 ± 0.066
	50	0.508 ± 0.081	0.491 ± 0.088	0.508 ± 0.079	0.494 ± 0.065	−0.00006 ± 0.041	0.547 ± 0.031
	100	0.512 ± 0.071	0.505 ± 0.068	0.512 ± 0.070	0.503 ± 0.044	0.004 ± 0.019	0.532 ± 0.028
RDC	10	0.522 ± 0.147	0.568 ± 0.227	0.528 ± 0.139	0.527 ± 0.143	0.062 ± 0.203	0.599 ± 0.038
	50	0.518 ± 0.085	0.522 ± 0.076	0.518 ± 0.083	0.515 ± 0.076	0.013 ± 0.039	0.551 ± 0.032
	100	0.517 ± 0.070	0.515 ± 0.042	0.517 ± 0.069	0.051 ± 0.04	0.007 ± 0.018	0.534 ± 0.026

**Figure 3:**
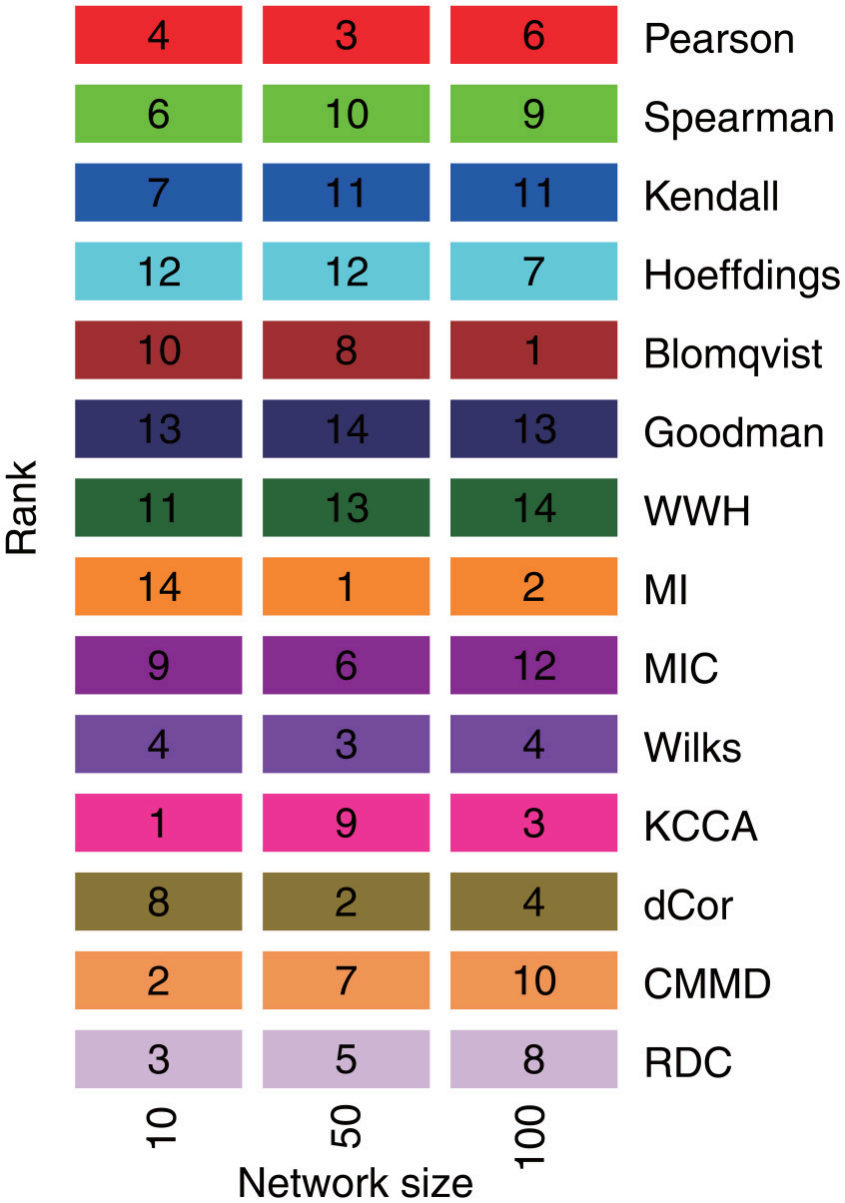
The ranks of 14 association measures in the inferences of regulatory networks with different node sizes. The numbers in the color blocks refer to the ranks of corresponding association measures by the means of AUC in these benchmark networks.

For the association measures, it becomes more difficult to achieve high inference performances when the network size becomes bigger from 10, 50 to 100. Although each association measure cannot achieve good inferences for big networks, the performances of them decrease with the same tendency. For 50-node networks, mutual information (MI) achieves the best AUC of 0.569 ± 0.046. Blomqvist's β performs the best for 100-node networks in the inference, while it is not stable for the small-size networks. Figure 3 shows the ranks of their performances according to the mean AUCs in different size of networks individually. From the comparative study, mutual information (MI) performs relatively better with stable ranks for big networks with 50 and 100 nodes. PCC is also stable in the 14 association measures for various sizes of network, as well as KCCA and dCor. This indicates their relative reliability in detecting gene regulatory relationships from expression data. For the other association measures, they accomplish unreliable and unstable regulatory network inferences in the benchmarks.

From the inference performances, we find that most of association-based methods can only achieve limited accuracies in the reconstruction of gene regulatory network from the benchmark datasets, especially for large-size networks. The application scopes of these association measures are mainly determined by the assumptions and characteristics of their definitions listed in Table 1. For instances, PCC is for linear regulatory relationship, MI is for non-linear relationship, KCCA and dCor measure the genuine relationship based on covariance, and the rank-based associations are robust to the noisy and outliers in gene expressions. In practical applications, the selection of suitable association measures could be subjectively determined by research purpose, experimental design, phenotypic condition and data quality. An ensemble and self-adaptive association measures selection strategy is desirable to be proposed for the co-existence of different gene regulatory relationships.

In real microarray data, we perform our comparative study of quantifying gene regulations during hepatitis C virus (HCV) infection on host Huh7 cells. The gene expression data are downloaded from NCBI GEO (accession ID GSE20948) (Edgar et al., 2002). There are 28 samples of 14 HCV infected Huh7 hepatoma cell samples and 14 corresponding mock-infected samples, originally designed three replicates at 6, 12, 18, 24, and 48 h post-infections, respectively. Two samples at 6 h have not been enrolled after quality control. The details can be accessed from Ref. (Blackham et al., 2010). We also download the hepatocellular carcinoma (HCC) gene set from KEGG (Kanehisa and Goto, 2000). The gene set contains 123 genes with 94 genes containing their expression profiles in GSE20948 (Edgar et al., 2002).

For evaluating the inference consistency of these association measures, we calculate the pairwise gene regulatory strengths in the HCC genes by the 14 association measures respectively. In the results of each association measure, the pairs with the top 5% association values are regarded as the identified gene regulations in the context of specific gene expression profiles after HCV infection.

**Figure 4:**
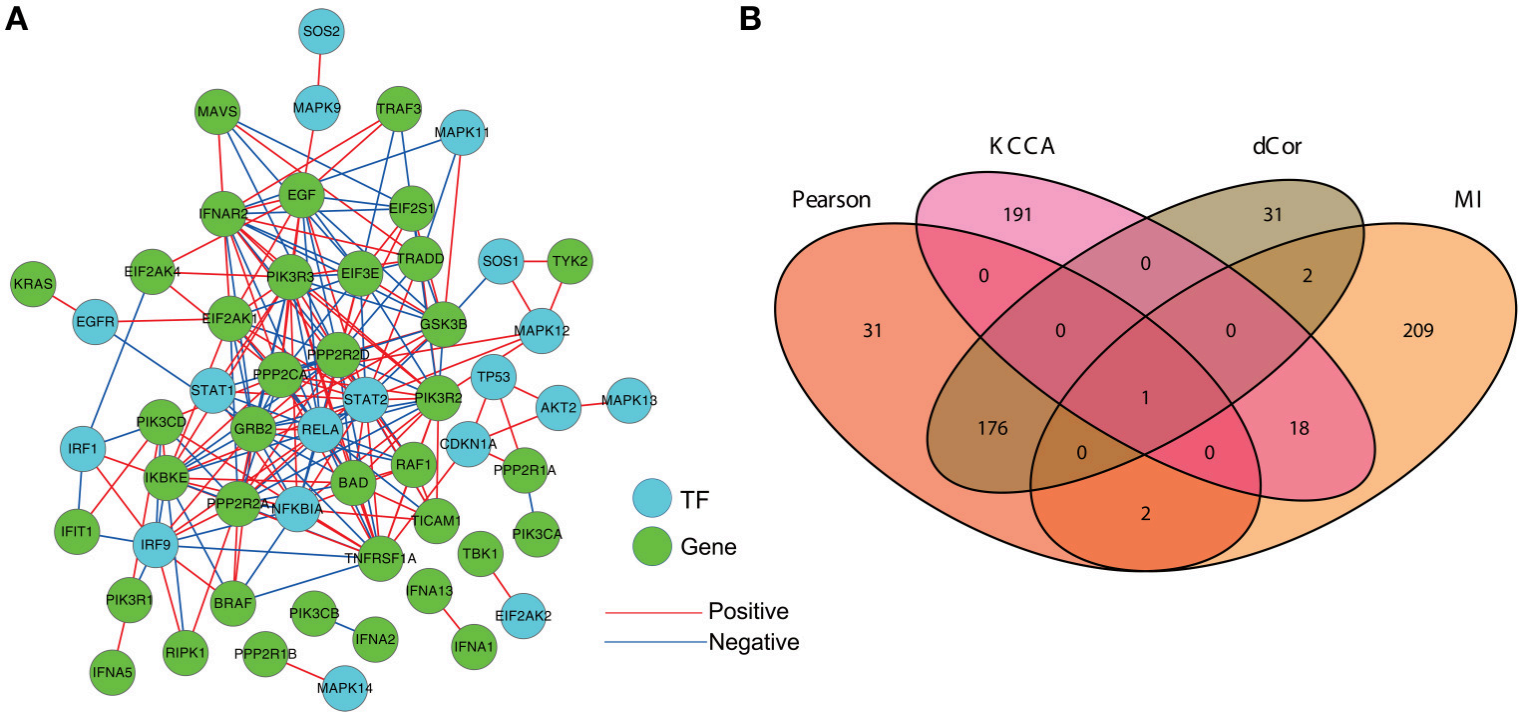
The reconstructed gene coexpression regulatory network during HCV infection. **(A)** The gene association network constructed by the PCC-based method. Isolated genes are not shown. **(B)** The overlapping status of the inferred gene regulations by four association measures, i.e., Pearson, MI, KCCA, and dCor.

Figure 4 demonstrates the inferred gene coexpression regulatory network in the HCC genes by PCC. There is no information about direction, so we annotate the known human TFs and display them by different color nodes (cyan) with the other genes (green). From Figure 4, we can figure out the regulatory information about positive and negative relationships during HCV infection. As in the former comparisons, we compare the overlapping status of these inferred coexpression relationships by the four association measures with top performances, i.e., Pearson, MI, KCCA and dCor. There exists only one pair of genes (“IFNA1” and “IFNA13”) is identified by the four measures, and the relationship between the two genes can be detected by any of them. Interestingly, Pearson and dCor contain many overlaps (177 regulations). It provides direct evidence that dCor is mainly to extract the linear correlations between genes as that Pearson done in this case study. There are few overlaps (3 regulations) between Pearson and MI, which indicates the linear and non-linear information are inconsistent with each other, and different association measures might identify different gene associations. The selection of suitable association measures is again proved to be very important for inferring gene coexpression regulatory network. The few overlapping regulations also imply the complex and diversity of regulatory relationships underlying gene expressions. More advanced methods beyond association measures are urged for elucidating gene regulatory mechanism from high-throughput data. See Section Discussion for some already available methods.

## Discussion

It is known association is different from causality and correlation does not imply causation (Altman and Krzywinski, 2015). Detecting the causality between genes has been essential in gene regulatory network inference since the availability of high-throughput data (Opgen-Rhein and Strimmer, 2007). Gene association network indicates more general gene-gene relationship than regulation, and gene regulatory network indicates more general gene-gene relationship than causality. The gene causality network, that is to say, the causal regulations between genes are directed in the gene-gene interaction graph with the detailed information of which ones are upstream regulators, and which ones are downstream targets. In the direct regulations, TFs or signal transductors causally affect their target gene expressions. The information flow transits between genes will be revealed if a causal relationship exists. So far, there is no association measure has been defined for describing the causal relationship between genes (Zhang et al., 2014; Zhao et al., 2016), while more advanced methods based on conditional probability, model-based regression and differential equation have been proposed to address the evaluations of causality.

Based on conditional independence, some improved association measures, such as partial correlation coefficient and conditional mutual information, have been proposed to eliminate false positive regulations from gene associations. The original association measures generate the footholds for detecting genuine relationships. Conditioning on another gene or gene set *Z*, partial correlation measure *r*_*XY*·*Z*_ between gene *X* and *Y* is to access the exact correlation between *X* and *Y* and that has no relationship with *Z* (de la Fuente et al., 2004). It is defined as

$$\begin{array}{l} r_{XY\cdot Z}=\frac{r_{XY}-r_{XZ}r_{YZ}}{\sqrt{\left(1-r_{XZ}^{2}\right)\left(1-r_{YZ}^{2}\right)}}. \end{array}$$

Where *r* refers to PCC. In the similar philosophy of introducing other gene or gene set, the conditional mutual information (CMI; Liang and Wang, 2008) is defined as

$$\begin{array}{l} I\left(X_{i},Y_{j}|Z_{k}\right)=\underset{X_{i}\in X,\;Y_{j}\in Y,\;Z_{k}\in Z}{\sum }p\left(X_{i},Y_{j},Z_{k}\right)\log\frac{p\left(X_{i},Y_{j}|Z_{k}\right)}{p\left(X_{i}|Z_{k}\right)p\left(Y_{j}|Z_{k}\right)}. \end{array}$$

Based on CMI and the order of conditioned gene numbers, we proposed a gene regulatory inference method named PCA-CMI (Zhang et al., 2012, 2013), which detect out dedicate associations by removing undirect false positive regulations. For a pair of genes *X* and *Y*, Li proposed a conditional coexpression measure named liquid association (LA) between two genes by introducing a third gene *Z* (Li, 2002). Based on *Z*, the gene relationship of *X* and *Y* is defined as

$$\begin{array}{l} LA\left(XY|Z\right)=E\left(XY|Z\right)=\underset{i}{\sum }\frac{X_{i}Y_{i}Z_{i}}{n} \end{array}$$

where *n* is the sample size. The LA activity determines the functional associations of gene *X* and *Y* in the condition of *Z*.

Currently, the causality between genes is often quantified via Bayesian models (Friedman et al., 2000). According to data, the conditional probability of $P\left(X|Y\right)=\frac{P\left(Y|X\right)P\left(X\right)}{P\left(Y\right)}.$ The probability of gene *X* conditioned on gene *Y*, means *Y* have a causal effect on *X* because there exists a negative or positive values of the conditional probability. The structured model has been extended and formulated as diagrams using a graphical criterion known as *d*-separation (Bareinboim and Pearl, 2016). Bayesian network provides a model-based detection of causal regulatory relationships. Gene regulations are then identified from the graphical models (Liu et al., 2013).

Regression and other structured models often extract the effects of regulatory coefficients. The identification of model coefficients determines the global relationship of these individual genes (D'Haeseleer et al., 1999). Specifically, the regression models the response gene as the linear combinations of the other dependent genes, i.e., *Y* = *c*_0_ + *c*_1_*X*_1_ + *c*_2_*X*_2_ + ⋯ + *c*_*m*_*X*_*m*_ + ε, *m* is the number of dependent genes in the regression and ε is the error variable. In generalized linear models, the response gene is changed to θ(*Y*), and *X*_1_, ⋯ , *X*_*m*_ are replaced by ϕ_1_(*X*_1_), ⋯ , ϕ_*m*_(*X*_*m*_), respectively (Breiman and Friedman, 1985). In the special case of simple linear regression with *m* = 1, the model is to detect the linear relationship between the response gene and the only one dependent gene. The coefficient of determination denoted by *r*^2^ is equal to the square of PCC (Altman and Krzywinski, 2016). The coefficient of determination, which represents the proportion of variation due to their linear relationship, generalizes the correlation coefficient for relationships beyond simple linear regression. Often, the regression equations often model the associations between response genes and dependent genes in an inter-coupled system. From a system biology perspective, regression models consider the genes in an integrated manner. Compared to the former pairwise associations, they identify more complicated relationships among genes. After determining the coefficients, the relationships in these genes are quantified correspondingly. How to determine crucial regulators and targets via statistical variable selections techniques, such as lasso (Tibshirani, 1996) and elastic net (Zou and Trevor, 2005), are substantially important.

Similarly, ODE models the derivatives, i.e., $\frac{dY}{dt}=c_{0}+c_{1}X_{1}+c_{2}X_{2}+\cdots +c_{m}X_{m}$, and so ODE quantifies the dynamics of the response as a function of the dependents in the system (Wu et al., 2014). The expression change rate of a response gene is modeled by the expressions of dependence genes. The *Y* might be another dependence gene and thus the system is closed. The system identification is to evaluate the coefficients in the right-hand side of the equation and the coefficient values refer to gene regulatory strengths. When the coefficient is 0, there is no relationship between the responding gene and the depending gene, otherwise the regulatory strength can be represented by positive or negative numeric values.

Compared to association measure, regression model and differential equation model regard gene regulatory network as an integrative system. The gene regulatory network inference is then transformed to a system identification problem of solving the coupled equations. The gene regulation strengths refer to the identified coefficients. From a sequential modeling perspective, the causality between regulators and targets can also be reflected by these system biology techniques.

In machine learning techniques such as clustering (Rui and Wunsch, 2005), there are some metrics have been developed for measuring the association between data points. The distances of Euclidean, cosine, Hamming, Manhattan are often used to measure gene relationships in gene expression clustering (D'Haeseleer, 2005). These distances evaluate the differences including dependences between genes, while these compared association measures focus on quantifying gene relationship such as regulation between genes. In gene expression data analyses of clustering and feature selection, distance metrics provide alternatives to define gene similarities. The distance metrics are not included in the comparative study for their diversity and case-intensity (Santini and Jain, 1999).

## Conclusions

In this paper, we summarized and compared the main proximities and metrics for quantifying gene regulatory associations. Written in full, the definitions and descriptions of 14 association measures are summarized and their characteristics with applications in regulatory network inference have been presented. From the benchmark challenge data and real gene expression data, we compared their performances and consistencies in the network inferences. Furthermore, their advantages and limitations are also analyzed and discussed. Currently, developing causality measure is an urgent research topic from driving gene association to regulation causality (Bareinboim and Pearl, 2016). A powerful measure of causality will greatly benefit the discovery of important gene regulations. Moreover, the linear/non-linear regression and differential equation models regard many genes in dynamic systems and the parameters of these models represent the system in details. The model-based gene regulatory network inference methods seem to provide more powerful tools when compared to the association-based methods. However, the association measures contain their flexibility in sense, easy interpretation and large scope of applications.

In conclusion, gene association measures provide fundamental quantifications of detecting gene regulatory relationships from transcriptomic profiling data. The high-throughput technologies advance the measurements of thousands of genes in parallel manners. The association measures effectively accelerate the transformation processes from data to knowledge. Most of the proposed association measures are statistical techniques which focus only on the inter-relationships between genes, and they are very hard to get the causal gene relationships alone. With the improved conditional or joint association measures, such as partial correlation coefficient, conditional mutual information and liquid association, the causality between genes can be partially extracted out from data. The introduction of other genes in evaluating gene regulation provides promising alternatives to grasp the genuine regulations. For an entire system, many genes perform their functions coordinately and cooperatively. So more advanced models are extremely needed to describe the complex system of gene regulations. In such model as ODE, the time-varying regulations are exactly to quantify the gene regulatory interactions with temporal implications. For the model complexity and the data availability, the dynamics underlying the coefficients in regression and ODE will reveal much more complicated regulatory relationships.

## Author contributions

ZL conceived and designed the study. ZL wrote the code and analyzed the data. ZL drafted the manuscript.

### Conflict of interest statement

The author declares that the research was conducted in the absence of any commercial or financial relationships that could be construed as a potential conflict of interest.
